# Immunotherapy-associated lichen planus pemphigoides successfully treated with intravenous immune globulin—Two illustrative cases

**DOI:** 10.1016/j.jdcr.2024.08.042

**Published:** 2024-09-20

**Authors:** Zachary Clayton Ney, Lowell T. Nicholson, Lauren M. Madigan

**Affiliations:** a Spencer Fox Eccles School of Medicine, University of Utah, Salt Lake City, Utah; b Department of Dermatology, University of Utah, Salt Lake City, Utah

**Keywords:** immunoglobulin, immunotherapy, IVIG, lichen planus pemphigoides, lichenoid dermatitis, PD-1 inhibitor

## Introduction

Lichen planus pemphigoides (LPP) is a rare, immune-mediated blistering disease with characteristics of both lichen planus (LP) and bullous pemphigoid (BP).^1^ LPP can be idiopathic or associated with a trigger such as a chronic viral infection or medication, including immune checkpoint inhibitors.^2^^,^^3^ Treatment is highly variable, and systemic therapies are often needed.^4^ Herein, we report 2 cases of treatment-refractory LPP secondary to immunotherapy that were successfully treated with intravenous immunoglobulin (IVIg) (Table I).

**Table I:** Patient characteristics

	Patient 1	Patient 2
Age, y	62	77
Sex	Female	Male
Oncologic diagnosis	Stage IIIB malignant melanoma	Stage IVB metastatic gastric adenocarcinoma
Time to rash onset	6 wk from initiation of pembrolizumab	9 mo from initiation of pembrolizumab
Cutaneous findings	Violaceous papules and vesicular plaques on the trunk and extremities, hemorrhagic-crusted erosions on the lips, erythema, and erosions on the palatal and buccal mucosa and posterior pharynx.	Hyperkeratotic lichenoid papules on the trunk and extremities with a large ulceration encompassing the dorsal and lateral aspects of the tongue
Biopsy H&E	Lichenoid drug reaction favored (hyperkeratosis, wedge-shaped hypergranulosis, with a dense, lichenoid inflammatory infiltrate, scattered eosinophils, subepidermal separation, and pigment incontinence).	Lichenoid drug reaction favored (compact hyperkeratosis with lichenoid inflammation, tissue eosinophils, and subepidermal cleft formation, as well as numerous Civatte bodies).
Biopsy DIF and IIF	Sharp linear deposition of Ig and complement along the basement membrane with elevated BP180 and BP230 antibodies	IgG basement membrane zone antibody reactivity with elevated BP180 and BP230 antibodies
Prior therapies	Corticosteroids, dapsone, methotrexate, hydroxychloroquine, apremilast, and dupilumab	Corticosteroids, infliximab, cyclosporine, methotrexate, hydroxychloroquine, and dupilumab
Current therapy	SCIg (transitioned from IVIg to improve tolerability), hydroxychloroquine, and apremilast	IVIg, methotrexate (actively tapering), and hydroxychloroquine
Clinical course	Full resolution of rash with only intermittent mild oral discomfort without lesions	Complete resolution of cutaneous and mucosal lesions

*BP180*, Bullous pemphigoid 180; *BP230*, bullous pemphigoid 230; *DIF*, direct immunofluorescence; *IG*, immunoglobulin G; *IIDF*, indirect immunofluorescence; *IVIg*, intravenous immunoglobulin; *SCIg*, subcutaneous immunoglobulin.

## Case 1

**Fig 1:**
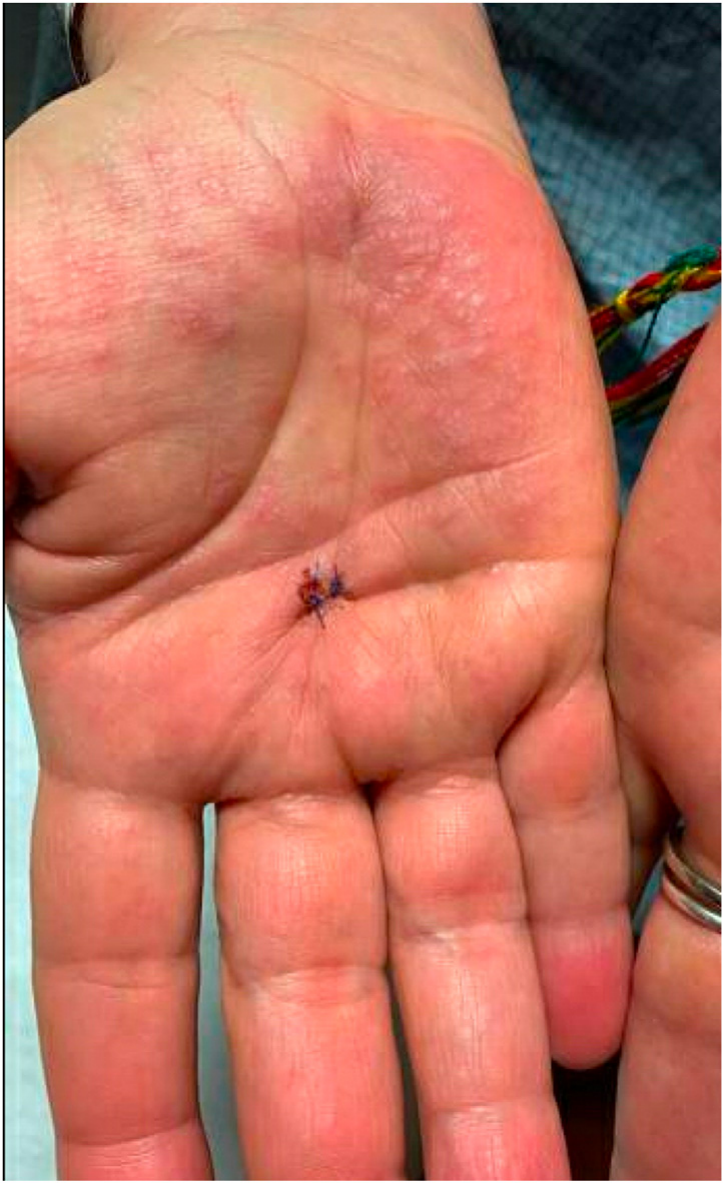
Representative image from Case 1 demonstrating an erythematous to violaceous plaque on the right palm with associated vesicular change.

A woman in her 60s with a history of stage IIIB melanoma was hospitalized with a diffuse rash and painful mucositis 6 weeks after initiation of pembrolizumab. Examination revealed numerous red to violaceous, flat, scaly papules and larger vesicular plaques, scattered on the trunk and extremities, including the palms and soles (Fig 1). Additionally, hemorrhagic-crusted erosions were noted on the lips, and erythema and erosions were seen on the palatal and buccal mucosa and posterior pharynx. Testing for herpes simplex virus and group A streptococcus was negative. A skin biopsy from the right hand revealed hyperkeratosis, wedge-shaped hypergranulosis, and a dense, lichenoid inflammatory infiltrate with scattered eosinophils, subepidermal separation, and pigment incontinence. A lichenoid cutaneous immune-related adverse event was favored, and systemic corticosteroids were initiated with initial improvement then subsequent recurrence upon tapering. Pembrolizumab was discontinued at this time without improvement. Biopsies were performed on the right buccal mucosa, which again revealed a pattern consistent with a lichenoid reaction comprised of a lymphocytic infiltrate obscuring the basal layer of the epidermis. Direct immunofluorescence showed sharp linear deposition of immunoglobulin (IgG, IgA) and complement (C3) along the basement membrane zone in addition to numerous cytoid bodies and fibrinogen. Serum studies revealed elevated BP180 and BP230 autoantibodies. Taken together, these findings were consistent with pembrolizumab-induced LPP. Over the next 18 months, multiple systemic therapies were trialed, including dapsone, methotrexate, hydroxychloroquine, apremilast, and dupilumab, all with minimal efficacy. More potent immunosuppressants were discouraged by oncology given gains achieved with prior immunotherapy. In this setting, she was started on monthly IVIg (2 gram/kg/month) in combination with hydroxychloroquine and apremilast (a combination that was previously ineffective alone). This resulted in gradual improvement and a significant reduction in pain. She was eventually transitioned to subcutaneous immune globulin for ongoing management, and she is currently experiencing only intermittent mild oral discomfort without mucocutaneous lesions and has no evidence of cancer recurrence.

## Case 2

**Fig 2:**
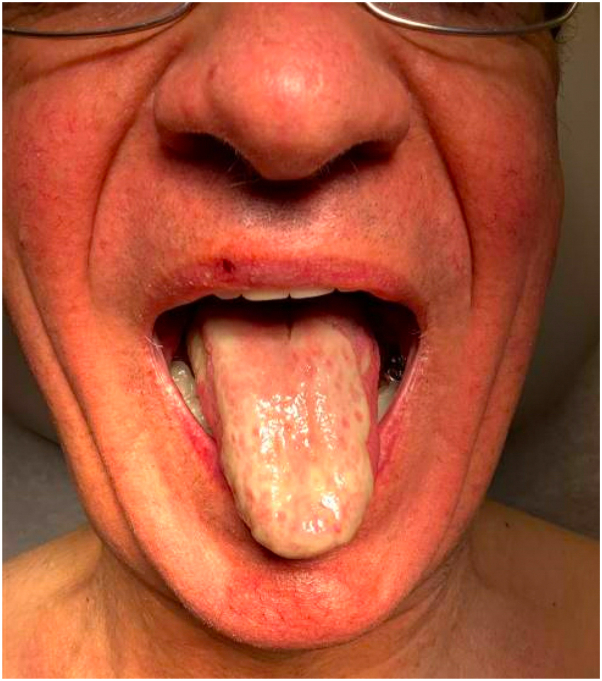
Representative image from Case 2 demonstrating a large oral ulceration encompassing the majority of the dorsal surface of the tongue.

A man in his 70s with a history of metastatic gastric adenocarcinoma was referred to dermatology for evaluation of a widespread rash and treatment of refractory oral ulcers, which appeared 9 months after starting pembrolizumab. Prior to referral, he had been managed by oncology and was treated with topical and systemic corticosteroids, as well as infliximab without improvement. Pembrolizumab had been discontinued. Examination demonstrated scattered hyperkeratotic lichenoid papules on the trunk and extremities and a broad ulceration covering the majority of the dorsal and lateral tongue (Fig 2). In this setting, he was unable to eat and required nutritional support. A skin biopsy of the right forearm revealed compact hyperkeratosis with lichenoid inflammation comprised predominantly of lymphocytes and also with many neutrophils and rare eosinophils. Subepidermal cleft formation was observed as well as numerous Civatte bodies. Indirect immunofluorescence demonstrated IgG basement membrane zone antibody reactivity on monkey esophagus substrate and epidermal localization on salt-split skin substrate with elevated BP180 and BP230 autoantibodies by enzyme-linked immunosorbent assay. A diagnosis of LPP was favored, and he was started on topical corticosteroids, cyclosporine swish and spit, methotrexate, and hydroxychloroquine. One month later, he reported some improvement in cutaneous lesions but was still experiencing significant oral disease. He was subsequently started on dupilumab but several months later reported persistent mouth sores, to the extent that he was having trouble with oral intake. Again, additional immunosuppression was discouraged by oncology given immunotherapy response. As such, IVIg was initiated in addition to low dose methotrexate and hydroxychloroquine (both of which had been utilized for >4 months without improvement in mucositis). This resulted in significant improvement in symptoms (Supplementary Fig 1, available via Mendeley at https://data.mendeley.com/datasets/6wf6ttc72x/1). At the time of this publication, he is without any cutaneous or mucosal lesions, and recent imaging is without any evidence of cancer recurrence. He is now tapering off of methotrexate without recurrence to date (though notably a delayed dose of IVIg resulted in a flare of oral pain and mucosal erythema, which resolved rapidly with resumption of therapy).

## Discussion

LPP is a rare autoimmune blistering disease characterized by overlapping clinical and pathologic features of LP and BP. Clinically, it manifests as widespread lichenoid papules and plaques, often accompanied by tense bullae and erosions, which may appear within pre-existing lichenoid lesions or on previously unaffected skin. Mucosal involvement may occur.^1^^,^^2^ Histopathologically, subepidermal blistering with a lichenoid infiltrate is often present and confirmatory studies classically demonstrate deposition of IgG/C3 along the basement membrane zone with positive BP180 and BP230 autoantibodies.^1^^,^^2^ LPP in association with immunotherapy tends to be delayed presenting after several weeks to months of therapy—with a reported range of onset from 1 week to 24 months (average 17-24.4 weeks).^3^^,^^4^ Most patients will experience involvement of the trunk and extremities with approximately 39% to 50% developing mucosal involvement per recent reviews.^2^^,^^3^

Treatment for LPP is highly variable, and an evidence-based treatment algorithm has not yet been established. First-line therapies typically include topical and systemic corticosteroids, but the disease can be refractory, and steroid-sparing therapies are often needed. A variety of systemic therapeutic agents have been employed, including hydroxychloroquine, dapsone, acitretin, methotrexate, azathioprine, mycophenolate, cyclosporine, dupilumab, and Janus kinase inhibitors.^4^^,^^5^ Treatment of patients with immune checkpoint inhibitor-induced LPP is especially challenging given a tendency toward persistence even when immunotherapy is discontinued, the risks associated with use of potent immunosuppressive agents in the setting of advanced malignancy, and the potential impacts on response to therapy.^2^^,^^3^

In this setting, IVIg may be a safe and attractive therapeutic option. Although the mechanism of action for IVIg is complex and not completely understood, it is not thought to be systemically immunosuppressive in a conventional sense. Within dermatology, uses of IVIg include dermatomyositis, toxic epidermal necrolysis, pyoderma gangrenosum, and autoimmune blistering disease, including pemphigus and pemphigoid variants.^6^ Reports also suggest that IVIg may be an efficacious adjuvant or second-line therapy for LP.^7^

As reported here, the refractory nature of cutaneous and oral lesions in our patients, in addition to a clinical need to avoid significant systemic immunosuppression, led to treatment with IVIg. For both patients, IVIg proved effective and resulted in a rapid improvement of symptoms, especially pain. Although improvement of cutaneous and oral lesions was more gradual, several months of therapy ultimately led to near-complete or complete resolution in both patients. It is notable that these individuals have also experienced a complete and sustained response to immunotherapy without evidence of recurrent malignancy, despite discontinuation of pembolizumab earlier than planned. In those who require ongoing therapy, additional questions regarding the impact of IVIg and timing of therapy would need to be considered. These findings highlight the use of IVIg as a potentially safe and effective treatment in patients with immune checkpoint inhibitor-induced LPP.

## Conflicts of interest

None disclosed.
